# Pleural effusion during weaning from mechanical ventilation: a prospective observational multicenter study

**DOI:** 10.1186/s13613-018-0446-y

**Published:** 2018-11-01

**Authors:** Keyvan Razazi, Florence Boissier, Mathilde Neuville, Sébastien Jochmans, Martial Tchir, Faten May, Nicolas de Prost, Christian Brun-Buisson, Guillaume Carteaux, Armand Mekontso Dessap

**Affiliations:** 1 AP-HP, DHU A-TVB, Service de Réanimation Médicale, Hôpitaux Universitaires Henri Mondor, 94010 Créteil, France; 2 Faculté de Médecine de Créteil, IMRB, GRC CARMAS, Université Paris Est Créteil, 94010 Créteil, France; 3 Unité U955 (Institut Mondor de Recherche Biomédicale), INSERM, Créteil, France; 4 Service de Réanimation Médicale, Centre Hospitalier Universitaire de Poitiers, Poitiers, 86021 France; 5 AP-HP, Service de Réanimation Médicale, Hôpital Européen Georges Pompidou, 75015 Paris, France; 6 AP-HP, Réanimation Médicale et des Maladies Infectieuses, Hôpital Bichat Claude Bernard, Paris, France; 7 Département de Médecine Intensive, Groupe Hospitalier Sud Ile-de-France, Hôpital de Melun, 77011 Melun, France; 8 Service de Réanimation, Centre Hospitalier de Villeneuve-Saint-Georges, 94190 Villeneuve-Saint-Georges, France

**Keywords:** Mechanical ventilation, Pleural effusion, Weaning, Ultrasonography

## Abstract

**Background:**

Pleural effusion is common during invasive mechanical ventilation, but its role during weaning is unclear. We aimed at assessing the prevalence and risk factors for pleural effusion at initiation of weaning. We also assessed its impact on weaning outcomes and its evolution in patients with difficult weaning.

**Methods:**

We performed a prospective multicenter study in five intensive care units in France. Two hundred and forty-nine patients were explored using ultrasonography. Presence of moderate-to-large pleural effusion (defined as a maximal interpleural distance ≥ 15 mm) was assessed at weaning start and during difficult weaning.

**Results:**

Seventy-three (29%) patients failed weaning, including 46 (18%) who failed the first spontaneous breathing trial (SBT) and 39 (16%) who failed extubation. Moderate-to-large pleural effusion was detected in 81 (33%) patients at weaning start. Moderate-to-large pleural effusion was associated with more failures of the first SBT [27 (33%) vs. 19 (11%), *p* < 0.001], more weaning failures [37 (47%) vs. 36 (22%), *p* < 0.001], less ventilator-free days at day 28 [21 (5–24) vs. 23 (16–26), *p* = 0.01], and a higher mortality at day 28 [14 (17%) vs. 14 (8%), *p* = 0.04]. The association of pleural effusion with weaning failure persisted in multivariable analysis and sensitivity analyses. Short-term (48 h) fluid balance change was not associated with the evolution of interpleural distance in patients with difficult weaning.

**Conclusions:**

In this multicenter observational study, pleural effusion was frequent during the weaning process and was associated with worse weaning outcomes.

**Electronic supplementary material:**

The online version of this article (10.1186/s13613-018-0446-y) contains supplementary material, which is available to authorized users.

## Introduction

Several factors may contribute to the occurrence of pleural effusions in critically ill patients, including heart failure, pneumonia, hypoalbuminemia, and fluid overload [1]. Its incidence in mechanically ventilated patients varies depending on the screening method, from approximately 8% with physical examination to more than 60% with routine ultrasonography [1, 2]. Pleural effusion was found in 83% of patients with acute respiratory distress syndrome (ARDS) explored with computed tomography scans [3].

The presence of pleural effusion is associated with a longer duration of mechanical ventilation and intensive care unit (ICU) stay [2]. Although a causal relationship cannot be established, this prolongation may result from altered respiratory mechanics [4] and impeded diaphragmatic contraction [5]. Indeed, pleural effusion increases the total thoracic volume, leading inspiratory muscles to operate in a less advantageous portion of their length-tension curve. Thus, the capacity of the diaphragm to generate pressure decreases when pleural effusion increases [5, 6]. Drainage of large pleural effusions improves oxygenation and respiratory mechanics in mechanically ventilated patients [4, 7].

Weaning accounts for approximately 40% of the total duration of mechanical ventilation [8], but data on pleural effusion during the weaning process are scarce [9]. The main objective of the present observational multicenter study was to assess the prevalence and risk factors of pleural effusion at initiation of weaning. The second objective was to explore the association of pleural effusion with weaning outcomes, and its evolution during difficult weaning.

## Materials and methods

This prospective multicenter observational study recruited patients admitted in five ICUs in France. Inclusion criteria were endotracheal mechanical ventilation for at least 24 h, and the fulfillment of weaning criteria [10] allowing a first spontaneous breathing trial (SBT). Noninclusion criteria were pregnancy or lactation, age less than 18 years, pleural effusion drainage before the first SBT, and a do-not-reintubate decision at time of inclusion.

### Weaning protocol and definitions

Weaning initiation was defined as the day of first SBT. The first SBT used a T-piece trial in three centers and a low-level pressure support (7–10 cm H_2_O) with zero end-expiratory pressure in two centers, as per usual care. Failure of the SBT was based on predefined criteria (see the online supplement, Additional file 1). Extubation failure was defined as death or reintubation within the 7 days following extubation; this delay was used instead of 48–72 h because prophylactic noninvasive ventilation may postpone reintubation [11]. Indications for prophylactic noninvasive ventilation included patients older than 65 years and those with underlying cardiac or respiratory disease [12]. According to the International Consensus Conference [10], weaning success was defined as a first successful SBT followed by successful extubation. Failure of the weaning process was defined [10] as failure of the first SBT or extubation failure. Because some patients could not be classified with this definition, weaning was also categorized according to the WIND definition [13] as follows: short when the first SBT resulted in a successful termination of the weaning process or death within 1 day after the first SBT; difficult in case of successful weaning or death after more than 1 day but in less than 1 week after the first SBT; prolonged if weaning was still not terminated 7 days after the first SBT. Ventilator-free days at day 28 were computed as days without invasive mechanical ventilation during the 28 days following first SBT; patients who died before day 28 or were dependent on mechanical ventilation for more than 28 days after the first SBT had zero ventilator-free days [14]. Other definitions (e.g., Mac Cabe classification, ARDS, ventilator-associated pneumonia, failure of SBT) and data collection process are reported in the online supplement (Additional file 1).

### Lung ultrasonography

Lung ultrasonography was performed on the day of first SBT and repeated on the 2 days following a SBT failure and on the day of extubation, if applicable. Maximal end-expiratory interpleural distance, sonographic patterns of effusion (homogeneously anechoic, complex nonseptated, complex septated, or homogeneously echogenic) [15], and of lung parenchyma (condensation or atelectasis) [16] were assessed on each side with the patient in the semirecumbent position. A moderate-to-large pleural effusion was defined as a maximal interpleural distance ≥ 15 mm (predicting an effusion volume of 300 mL or more) [17]; a large pleural effusion was defined by a maximal interpleural distance ≥ 25 mm [4, 17]. A pleural effusion was deemed drainable if the maximal interpleural distance was ≥ 15 mm, and the effusion was visible over at least three intercostal spaces [18]. When possible, a transthoracic echocardiography was also performed to assess left ventricle ejection fraction (see the online supplement, Additional file 1). In patients with SBT failure, attempts at depletion (by diuretics or ultrafiltration) and fluid balance were collected during the 2 days following the SBT. There was no mandatory depletive strategy for the management of pleural effusion.

### Statistical analysis

The primary endpoint was the prevalence of pleural effusion at weaning start. The sample size was calculated by hypothesizing a prevalence of pleural effusion of 40% [1–3], and considering a precision of 8%. The study required a minimum of 170 patients (for an alpha risk of 5%, i.e., a confidence interval of 95%) and a maximum of 260 patients (for an alpha risk of 1%, i.e., a confidence interval of 99%). Continuous data were expressed as medians [25th–75th centiles] unless otherwise specified, and were compared using the Mann–Whitney test. Categorical variables, expressed as percentages, were compared using the Chi-square test or Fisher exact test. To evaluate independent factors associated with the presence of moderate-to-large pleural effusion at weaning start or with failure of the weaning process, significant or marginally significant (*p* < 0.10) bivariate risk factors (using the above mentioned tests) were examined using univariate and multivariable backward stepwise logistic regression analysis. Among related univariate factors, only the most statistically robust (yet clinically relevant) was entered into the regression model in order to minimize the effect of colinearity. The selection process was guided by consistency (less than 5% missing values) and maximal imbalances between groups (as estimated by absolute standardized differences, which are independent of the sample size and variable unit) [19]. Coefficients were computed by the method of maximum likelihood. The calibration of models was assessed by the Hosmer–Lemeshow goodness-of-fit statistic (good fit was defined as *p* value > 0.05), and discrimination was assessed by the area under the receiver operating characteristics curve (with a value of 1 indicating perfect discrimination, and a value of 0.5 indicating the effects of chance alone). Correlations were tested using the Spearman’s method. Two-tailed *p* values < 0.05 were considered significant. Data were analyzed using the IBM SPSS Statistics for Windows (Version 19.0, IBM Corp Armonk, NY, USA).

## Results

### Study population

**Fig. 1:**
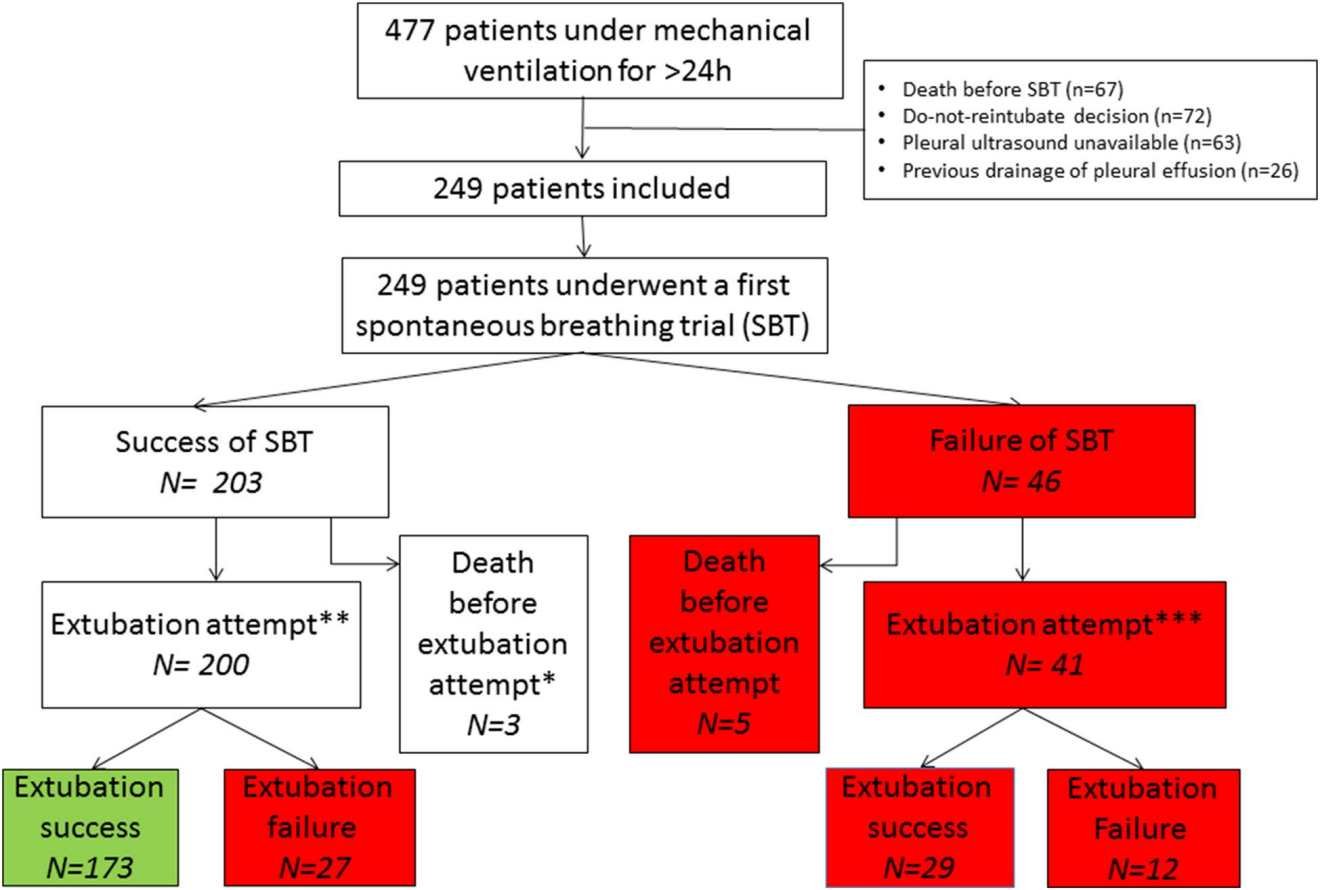
Study flow chart; green and red squares denote International Consensus Conference classification of weaning success and failure, respectively; *three patients were not extubated despite the success of the first SBT because of borderline cough, and experienced a novel fatal complication leading to death before any extubation attempt; they could not be classified according to the International Consensus Conference **including 192 planned and 7 unplanned. *** Including 39 planned and 2 unplanned

The inclusion period lasted from 2 to 12 months depending on centers, between June 2015 and May 2016. Four hundred seventy-seven patients mechanically ventilated for more than 24 h were screened (Fig. 1). Sixty-seven patients died before weaning start, and 161 patients were excluded because of either a do-not-reintubate decision at time of inclusion (*n* = 72), unavailability of pleural ultrasound (*n* = 63), or drainage of pleural effusion before inclusion (*n* = 26). Thus, the present study comprises 249 patients assessed with lung ultrasonography at weaning initiation. Median duration of mechanical ventilation before weaning was 4 [2–7] days. The weaning trajectories are summarized in Fig. 1. Two hundred and three patients succeeded the first SBT, and 200 of them were extubated (the remainder three patients were not extubated despite the success of the first SBT because of borderline cough, and experienced a novel complication leading to death before any extubation attempt). Forty-six patients (18%) failed the first SBT; 41 of them succeeded a subsequent SBT and were extubated latter in the course of weaning, while the remainder five patients died before any extubation attempt. Reasons for SBT failure were respiratory rate > 35 breaths/min with increased accessory muscle activity (*n* = 17), SpO_2_ < 90%, while on FiO_2_ ≥ 0.5 (*n* = 6), systolic blood pressure < 90 mmHg or > 180 mmHg (*n* = 2), or a combination of those reasons (*n* = 22).

Overall, 241 patients were extubated during the weaning process, while eight patients died before any extubation attempt. Among the 241 patients extubated, 232 were planned and nine unplanned (including one accidental and eight self-extubations). After extubation, 95 (40%) patients received noninvasive ventilation prophylactically, while twelve (5%) received it for post-extubation acute respiratory failure. A total of 39 (16%) patients failed extubation. The main reason for reintubation was acute respiratory failure (*n* = 23, 73%).

### Prevalence and risk factors for pleural effusion

A moderate-to-large pleural effusion was detected in 81 of 249 patients assessed at weaning initiation, for a prevalence of 33%, 95% confidence interval: 27–39%. Most of pleural effusions were homogeneously anechoic (*n* = 74, 93%) and associated with pulmonary condensation or atelectasis (*n* = 68, 85%) (see the online supplement, Table e1, Additional file 1). Seventy-six (31%) patients had a bilateral pleural effusion. The maximal interpleural distance was equally located either on the left (*n* = 41, 51%) or right side (*n* = 40, 49%). Patients with moderate-to-large pleural effusions at weaning initiation were older, had more baseline comorbidities and more organ failures before weaning as compared to their counterparts (see the online supplement, Table e4). In multivariable analysis, older age, McCabe class 2, cardiac disease, acute respiratory failure as cause of intubation, and need for dialysis before the first SBT were the five independent factors associated with a moderate-to-large pleural effusion at initiation of weaning (Table 1).

**Table 1:** Univariate and multivariable analysis of factors associated with moderate-to-large pleural effusion

Variables	Missing values, *n* (%)	Absolute standardized differences	Odd ratio (95% confidence interval), *p* value by logistic regression	
			Univariate	Multivariable
Age (per year)	0	60.5	1.04 (1.02–1.06), *p* < 0.001	1.03 (1.01–1.05), *p* = 0.017
SAPS II (per point)	0	26.9	1.02 (1.0–1.03), *p* = 0.048	I/NR
Mc Cabe class II (yes vs. no)	0	51.8	4.7 (2.1–10.3), *p* < 0.001	4.2 (1.8–9.9), *p* = 0.001
Cancer or hematological malignancy (yes vs. no)	0	47	3.5 (1.7–7.2), *p* = 0.001	NI
Cardiac disease (yes vs. no)	0	55.1	3.3 (1.1–3.3), *p* < 0.001	2.2 (1.1–4.4), *p* = 0.02
Left ventricle ejection fraction at cardiac ultrasound (%),	44 (18%)	52.9	0.96 (0.93–0.98), *p* < 0.001	NI
Supra-ventricular arrhythmias (yes vs. no)	0	35.8	2.3 (1.3–4.2), *p* = 0.007	NI
Acute respiratory failure as cause of intubation (yes vs. no)	0	31.9	1.9 (1.3–3.9), *p* = 0.02	1.8 (0.98–3.2), *p* = 0.059
Dialysis (yes vs. no)	0	31.5	2.5 (1.2–5.4), *p* = 0.02	2.0 (0.9–4.6), *p* = 0.088
Serum Creatinine (per µmol/L)	0	25.8	1.0 (0.99–1.00), *p* = 0.20	NI
Septic shock (yes vs. no)	0	25.4	1.7 (0.98–2.9), *p* = 0.06	I/NR
ARDS (yes vs. no)	0	22.2	1.7 (0.91–3.1), *p* = 0.098	I/NR
Duration of MV before first SBT (per day)	0	19	1.04 (0.99–1.1), *p* = 0.15	NI

*SAPS II* simplified acute physiology score, *COPD* chronic obstructive pulmonary disease, *ARDS* acute respiratory distress syndrome, *SBT* spontaneous breathing trial, *NI* not included, *I/NR* included, but not retained by the final model

Among related univariate factors, only the most statistically robust (yet clinically relevant) was entered into the regression model in order to minimize the effect of colinearity. The selection process was guided by consistency (less than 5% missing values) and maximal imbalances between groups (as estimated by absolute standardized differences) as follows: Mc Cabe class II was selected among Mc Cabe class II, cancer and hematological malignancy; dialysis was selected among creatininemia and dialysis; cardiac disease was selected among supra-ventricular arrhythmias, left ventricle ejection fraction and cardiac disease; ARDS was selected among duration of mechanical ventilation before the first spontaneous breathing trial and ARDS before inclusion. The multivariable model showed a good calibration as assessed by the Hosmer and Lemeshow goodness-of-fit test [*χ*^2^ (8 d*f*) = 6.42, *p* = 0.60] and a fair discrimination as assessed by the receiver operating characteristics curve [area under the curve of 0.74 (0.67–0.80), *p* < 0.001]

### Outcome of weaning

**Fig. 2:**
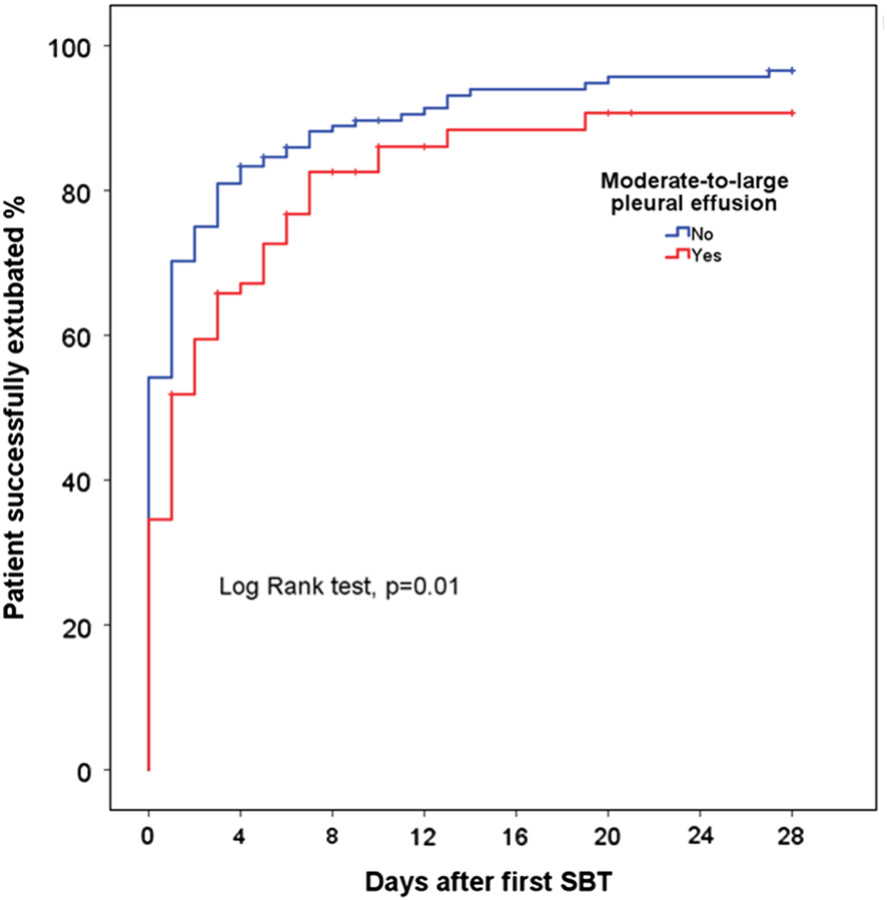
Kaplan–Meier curves for the probability of successful extubation after the first spontaneous breathing trial in mechanically ventilated patients with (red lines) or without (blue lines) moderate-to-large pleural effusion

According to the International Consensus Conference, the 249 patients were classified as follows: 173 (69%) weaning successes (a first successful SBT followed by successful extubation); 76 (31%) weaning failures (including 46 who failed the first SBT and 27 who succeeded the first SBT but failed extubation); three unclassifiable patients (despite the success of the first SBT, they were not extubated because of borderline cough, and experienced a novel complication leading to death before any extubation attempt) (Fig. 1). According to the WIND classification, 161 (65%) patients had a short weaning, 60 (24%) had a difficult weaning, and 28 (11%) had a prolonged weaning. The presence of a moderate-to-large pleural effusion at weaning initiation was associated with more failures of the first SBT [27 (33%) vs. 19 (11%), *p* < 0.001], more weaning failures [37 (47%) vs. 36 (22%), *p* < 0.001], less ventilator-free days at day 28 (21 [5–24] vs. 23 [16–26], *p* = 0.01), and a higher mortality at day 28 [14 (17%) vs. 14 (8%), *p* = 0.04] (Table 2, Fig. 2). All variables associated with weaning failure are shown in Table 3 and Table e5. In multivariable analysis, PaO_2_/FiO_2_ ratio, chronic obstructive pulmonary disease, a longer duration of mechanical ventilation prior to weaning, and the presence of moderate-to-large pleural effusion at weaning initiation were the four independent factors associated with weaning failure (Table 4). In sensitivity analyses, the association of pleural effusion with weaning failure also persisted after adjustment on SAPS II, in selected centers using the T-piece trial, or in those using a low-level pressure support, and when considering pleural effusions deemed drainable (as defined by a maximal interpleural distance ≥ 15 mm with the effusion visible over three intercostal spaces) or those considered large (as defined by a maximal interpleural distance ≥ 25 mm) [4, 17] (Table e3). A moderate-to-large pleural effusion was detected in 60 (28%) of 218 patients assessed on the day of first extubation attempt. The extubation failure rate was higher in patients with a moderate-to-large pleural effusion on the day of extubation as compared to their counterparts [14 (23%) vs. 19 (12%), *p* = 0.04]; of note, this extubation failure rate was similar in patients with or without pleural effusion assessed earlier, at weaning initiation [24 (15%) vs 15 (20%), *p* = 0.31]. As compared to patients without effusion (*n* = 168), those with a unilateral (*n* = 21) or bilateral (*n* = 60) moderate-to-large pleural effusion had similarly altered weaning outcomes, including SBT failure [19 (11.3%) vs. 8 (38.1%) vs. 19 (31.7%), *p* < 0.001] and weaning failure [37 (22.0%) vs. 11 (52.4%) vs. 28 (46.7%), *p* < 0.001].

**Table 2:** Characteristics and outcome of 249 mechanically ventilated patients with or without moderate-to-large pleural effusion at first spontaneous breathing trial

Variables	Moderate-to-large pleural effusion		*p* value
	Absent (*n* = 168)	Present (*n* = 81)	
Male gender	98 (58%)	52 (64%)	0.38
Age (years)	61 [50–72]	69 [60–80]	< 0.001
SAPS II score at ICU admission	49 [37–62]	52 [41–67]	0.07
*Comorbidities*			
Neurological disease	22 (13%)	6 (7%)	0.18
Cardiac disease	93 (55%)	65 (80%)	< 0.001
Cirrhosis	12 (7%)	9 (11%)	0.29
Chronic renal failure	22 (13%)	16 (20%)	0.17
Cancer or hematological malignancy	16 (10%)	22 (27%)	< 0.001
*Main reason for intubation*			
Coma	54 (32%)	9 (11%)	< 0.001
Acute respiratory failure	51 (30%)	37 (46%)	0.02
Septic shock	22 (13%)	12 (15%)	0.71
Others	41 (24%)	23 (28%)	0.5
*From ICU admission to first SBT*			
ARDS	32 (19%)	23 (28%)	0.096
Duration of MV before the first SBT	4 [2–7]	4 [3–9]	0.09
Dialysis	15 (9%)	16 (20%)	0.015
*Biological and ultrasound data at first SBT*			
Serum creatinine (µmol/L)	74 [55–119]	90 [60–164]	0.07
Serum protide (mg/L)	59 [54–66]	59 [51–63]	0.19
Bilateral pleural effusion	16 (10%)	60 (74%)	< 0.001
Maximal interpleural distance (mm)	0 [0–5]	27 [20–41]	< 0.001
Condensation or atelectasis of lung adjacent to the pleural effusion at ultrasound	–	68 (84%)	
Left ventricle ejection fraction (%)	60 [50–60]	50 [39–60]	< 0.001
*Outcome*			
Pleural effusion drainage during weaning	0	4 (5%)	0.005
Prophylactic NIV post-extubation	62 (38%)	33 (43%)	0.39
Failure of the first SBT	19 (11%)	27 (33%)	< 0.001
Extubation failure	24 (15%)	15 (20%)	0.31
Weaning failure^a^	36 (22%)	37 (47%)	< 0.001
Weaning group^b^			0.03
Short weaning	118 (70%)	43 (53%)	
Difficult weaning	38 (20%)	26 (32%)	
Prolonged weaning	16 (10%)	12 (15%)	
Tracheotomy	4 (2%)	2 (3%)	0.97
VFD from first SBT to day 28 (days)	23 [16–26]	21 [5–24]	0.01
Death in ICU	14 (8%)	13 (16%)	0.07
Death at day 28	14 (8%)	14 (17%)	0.04

Values are indicating number (%) or median [1st–3rd quartile]

^a^According to the international conference consensus (three patients could not be classified)

^b^According to the WIND study classification

*SAPS II* simplified acute physiology score, *ARDS* acute respiratory distress syndrome, *SBT* spontaneous breathing trial, *NIV* noninvasive ventilation, *ICU* intensive care unit, *VFD* ventilator-free days

**Table 3:** Variables associated with weaning failure in 246 mechanically ventilated patients (three patients could not be classified according to the international conference consensus definition)

Variables	Weaning success (*n* = 173)	Weaning failure (*n* = 73)	*p* value
Male gender	98 (57%)	49 (67%)	0.13
Age (years)	61 [52–73]	69 [60–79]	0.006
Body mass index (kg/m^2^)	26 [22–29]	27 [22–32]	0.07
SAPS II at ICU admission	49 [38–62]	49 [39–65]	0.73
*Comorbidities*			
COPD	23 (13%)	23 (32%)	0.001
Cardiac disease	101 (58%)	55 (75%)	0.01
*Main reason for intubation*			
Coma	54 (31%)	9 (12%)	0.002
Acute respiratory failure	48 (27%)	39 (53%)	< 0.001
Septic shock	24 (14%)	8 (11%)	0.54
Others	47 (27%)	17 (23%)	0.53
*From ICU admission to first SBT*			
ARDS	28 (16%)	27 (37%)	< 0.001
Neuromuscular blockade	26 (15%)	28 (38%)	< 0.001
Septic shock	61 (35%)	39 (53%)	0.01
VAP	17 (10%)	16 (22%)	0.01
Supra-ventricular arrhythmias	32 (19%)	22 (30%)	0.04
Duration of MV before first SBT	3 [2–6]	6 [3–12]	< 0.001
Dialysis	22 (13%)	8 (11%)	0.70
Fluid balance between ICU admission and first SBT (L)	2.8 [0.9–6.4]	5.7 [0.7–11.4]	0.01
*Biological and ultrasound data at first SBT*			
PaO_2_/FiO_2_ ratio (mmHg)	307 [242–385]	247 [200–299]	< 0.001
Moderate-to-large pleural effusion	42 (24%)	37 (51%)	< 0.001
Drainable pleural effusion	36 (21%)	29 (40%)	0.002
Large pleural effusion	21 (12%)	25 (34%)	< 0.001
Left ventricle ejection fraction (%, *n* = 205)	60 [50–60]	55 [40–60]	0.06
*Outcome*			
Pleural effusion drainage during weaning	0	4 (6%)	0.01
Prophylactic NIV post-extubation	65 (38%)	30 (44%)	0.35
Tracheotomy	1 (1%)	5 (7%)	0.01
VFD from first SBT to day 28 (days)	23 [20–26]	11 [0–21]	< 0.001
Death in ICU	5 (3%)	19 (26%)	< 0.001
Death at day 28	8 (5%)	17 (23%)	< 0.001

Values are indicating number (%), or median [1st–3rd quartile]

*SAPS II* simplified acute physiology score, *COPD* chronic obstructive pulmonary disease, *ARDS* acute respiratory distress syndrome, *VAP* ventilator-associated pneumonia, *SBT* spontaneous breathing trial, *NIV* noninvasive ventilation, *ICU* intensive care unit, *VFD* ventilator-free days

**Table 4:** Univariate and multivariable logistic regression of factors associated with weaning failure (n = 246)

Variables	Missing values, n (%)	Absolute standardized differences	Odd ratio (95% confidence interval), *p* value by logistic regression	
			Univariate	Multivariable
Age (per year)	0	47	1.03 (1.01–1.05), *p* = 0.01	1.02 (0.997–1.05), *p* = 0.08
Body mass index (per kg/m^2^)	6 (2%)	32	1.06 (1.01–1.11), *p* = 0.02	I/NR
COPD (yes vs. no)	0	48	3.0 (1.6–5.8), *p* = 0.001	2.2 (1.02–4.7), *p* = 0.045
Cardiac disease (yes vs. no)	0	37	2.2 (1.2–4.0), *p* = 0.01	I/NR
Left ventricle ejection fraction at cardiac ultrasound (%)	44 (18%)	27	0.98 (0.96–1.0), *p* = 0.09	NI
Supra–ventricular arrhythmias (yes vs. no)	0	26	1.9 (1.01–3.6), *p* = 0.046	NI
Septic shock (yes vs. no)	0	37	2.1 (1.2–3.7), *p* = 0.01	I/NR
Fluid balance between ICU admission and first SBT (per L)	15 (6%)	44	1.07 (1.03–1.12), *p* = 0.002	NI
Acute respiratory failure as cause of intubation (yes vs. no)	0	55	3.0 (1.7–5.2), *p* < 0.001	NI
PaO_2_/FiO_2_ ratio (per mmHg)	3 (1%)	58	0.994 (0.991–0.997), *p* < 0.001	0.996 (0.993–1.0), *p* = 0.03
Duration of MV before the first SBT (per day)	0	57	1.11 (1.06–1.17), *p* < 0.001	1.11 (1.05–1.17), *p* < 0.001
ARDS before the first SBT (yes vs. no)	0	49	3.0 (1.6–5.7), *p* < 0.001	NI
Neuromuscular blockade before the first SBT (yes vs. no)	0	54	3.5 (1.9–6.6), *p* < 0.001	NI
VAP before the first SBT (yes vs. no)	0	33	2.6 (1.2–5.4), *p* = 0.01	NI
Moderate-to-large pleural effusion (yes vs. no)	0	58	3.2 (1.8–5.7), *p* < 0.001	3.0 (1.5–5.8), *p* = 0.001

*SAPS II* simplified acute physiology score, *COPD* chronic obstructive pulmonary disease, *ARDS* acute respiratory distress syndrome, *VAP* ventilator-associated pneumonia, *SBT* spontaneous breathing trial, *NI* not included, *I/NR* included, but not retained by the final model

Among related univariate factors, only the most statistically robust (yet clinically relevant) was entered into the regression model in order to minimize the effect of colinearity. The selection process was guided by consistency (less than 5% missing values) and maximal imbalances between groups (as estimated by absolute standardized differences), as follows: cardiac disease was selected among supra-ventricular arrhythmias, left ventricle ejection fraction and cardiac disease; septic shock was selected among fluid balance between ICU admission and first SBT and septic shock; PaO_2_/FiO_2_ ratio was selected among acute respiratory failure as cause of intubation and PaO_2_/FiO_2_ ratio; duration of MV before the first SBT was selected among neuromuscular blockade, duration of MV before the first SBT, VAP, and ARDS. The multivariable model showed a good calibration as assessed by the Hosmer and Lemeshow goodness-of-fit test [*χ*^2^ (8 d*f*) = 6.8, *p* = 0.56] and a fair discrimination as assessed by the receiver operating characteristics curve [area under the curve of 0.76 (0.69–0.82), *p* < 0.001]

### Evolution of pleural effusion during difficult weaning

Among the 46 patients who failed the first SBT, lung ultrasonography was repeated 24 and 48 h later in 41 and 31 patients, respectively. Patients in whom diuretics and/or ultrafiltration were used had a lower fluid balance as compared with their counterparts (− 484 [− 1210–330] vs. 858 [205–1806] mL after 24 h, *p* < 0.001), but this depletive strategy did not alter the interpleural distance (see the online supplement, Table e2, Additional file 1). Fluid balance was not significantly correlated with changes in interpleural distance (*ρ* 0.13, *p* = 0, 17, see Figure e1 of the online supplement, Additional file 2). Pleural effusion was drained in only four patients during weaning.

## Discussion

We herein report the largest study assessing pleural effusion during weaning from mechanical ventilation. A moderate-to-large pleural effusion was detected by ultrasound examination in one-third of 249 patients at initiation of weaning and was associated with weaning failure by multivariable analysis. Depletive strategies did not alter pleural effusion volume on the short term in patients with difficult weaning.

### Prevalence and risk factors for pleural effusion

In our study, one-third of patients had a moderate-to-large pleural effusion at the initiation of the weaning process. This prevalence is higher than that of 13% reported by Dres et al. [9]. This discrepancy may be explained by differences in definitions used. Volume of pleural fluid was estimated in our report according to interpleural distance, which may be more sensitive than the classification of the British Thoracic Society [20] used in the latter study; indeed, patients with moderate-to-large pleural effusion in our study had a median interpleural distance inferior to the value found in the Dres’ study (27 [20–41] vs. 45 [30–60] mm). Other differences between these two studies include patient’s comorbidities (with more patients included with cardiac diseases in our report) and/or timing of inclusion (with less patients excluded because of prior pleural drainage before SBT in our study).

Risk factors for pleural effusion found in our study are in accordance with previous reports [1, 2]. Congestive heart failure is one of the leading factors associated with the occurrence of pleural effusion in ICU [1]. All patients intubated for acute respiratory failure had acute cardiac failure or pneumonia, two common risk factors for pleural effusion [1]. Our study suggests that diastolic dysfunction may be of importance in the association of cardiac failure with pleural effusion. Acute renal failure has also been previously reported as a risk factor for nonmalignant pleural effusions, an association possibly mediated by fluid overload [21]. The association of Mc Cabe class (i.e., a rapidly fatal underlying disease) with pleural effusion may be driven by other comorbidities like liver cirrhosis, cancer, and hypoalbuminemia [22, 23].

### Pleural effusion and weaning outcomes

Our study is the first to show an association between moderate-to-large pleural effusion on the one hand and worse weaning outcomes and survival on the other hand. Dres et al. found similar weaning outcomes in patients with or without pleural effusion, but the limited number of patients with pleural effusions in their report (*n* = 18) weakened their conclusions [9]. Our findings are consistent with a previous report by Mattison et al., suggesting an association between pleural effusion and a longer duration of mechanical ventilation [2]. Although pleural effusion in patients with early ARDS do not seem to significantly influence lung physiology and gas exchange [3], its role in the latter stages of mechanical ventilation seems more relevant [4]. Physiological alterations associated with pleural effusion may worsen the respiratory load during the weaning process. Indeed, physiological studies showed that pleural effusion increases chest wall volume and decreases the length of inspiratory muscles and thus their efficiency and power [5, 6]. Umbrello et al. recently showed that during weaning, drainage of a unilateral pleural effusion improves diaphragmatic contractile activity [24]. This improvement could decrease dyspnea and mitigate weaning failure. Pleural fluid accumulation may also result in relaxation atelectasis of the adjacent lung. In our study, most pleural effusions (85%) were associated with condensation or atelectasis.

### Clinical implications

There was no significant association between fluid balance and the evolution of pleural effusion during the 48 h following SBT failure. However, the negative fluid balance achieved was modest in the depletive group and this limitation precludes any definite conclusion. Pleural drainage with ultrasonography guidance has a low risk of complication under mechanical ventilator support [7] and may improve oxygenation, respiratory mechanics [4], and diaphragm performance [6, 24]. Removal of pleural fluid may therefore decrease the work of breathing and increase the ability of patients to succeed weaning. Further studies are needed to test whether a strategy of aggressive diuretic management or drainage of pleural effusions, in mechanically ventilated patients entering the weaning process, with others risk factors of weaning failure or a SBT failure, has the potential to decrease its duration [25].

### Strengths and limitations

Strengths of our study include the large sample size, the prospective and multicentric design, and the use of ultrasound, which is currently considered the most sensitive method to detect pleural effusion at bedside. Our study has several limitations. First, only 249 of the 477 screened patients were included, a fact that may alter the external validity of our prevalence estimation. Second, the inter- or intra-observer agreement for pleural ultrasonography was not evaluated, but several reports previously demonstrated an excellent agreement for the measurement of left or right maximal interpleural distance [26]. Third, the physicians in charge of the patient were not fully blinded to the ultrasound examination results, and this may have theoretically influenced extubation decision and outcomes. However, criteria for SBT result were defined a priori and independent from ultrasound findings. Fourth, no estimation of respiratory drive (e.g., with airway occlusion pressure) nor respiratory muscle strength (e.g., with maximal inspiratory pressures) was performed. Last, a significant decrease in pleural effusion during difficult weaning may have required more time, and/or more intense depletive fluid management [27].

## Conclusion

Moderate-to-large pleural effusion was found in one-third of patients at initiation of weaning and associated with worse outcomes. Depletive strategies did not rapidly alter its evolution. Further studies should test the clinical usefulness and safety of reducing moderate-to-large pleural effusion at initiation of ventilator weaning, either by aggressive depletion or drainage.

## Additional files

**Additional file 1.** Methods Supplement,  Table e1, Table e2, Table e3, Table e4, Table e5.

**Additional file 2.** Change in interpleural distance during the 24 and 48 h following failure of spontaneous breathing trial  according to fluid balance.

## Abbreviations

SBT: spontaneous breathing trial
ARDS: acute respiratory distress syndrome
ICU: intensive care unit
