# Supplementary material for: Pleural effusion during weaning from mechanical ventilation: a prospective observational multicenter study
Source: Ann Intensive Care. 2018 Nov 1;8:103. doi: 10.1186/s13613-018-0446-y (PMC6211142; doi:10.1186/s13613-018-0446-y)
Supplement: Supplementary file 1 — Additional file 1. Methods Supplement, Table e1, Table e2, Table e3, Table e4, Table e5. [file 13613_2018_446_MOESM1_ESM.docx]

**Online supplement:**

**Prevalence and impact of pleural effusion during weaning from mechanical ventilation: a prospective multicenter study**

Keyvan Razazi^1,2^, MD, Florence Boissier^3,4^, MD, PhD, Mathilde Neuville^5^, MD, Sébastien Jochmans^2,6^, MD, Martial Tchir^7^, MD, Faten May^1,2^, MD, Nicolas de Prost^1,2^ ,MD, PhD, Christian Brun-Buisson^1,2^, MD, Guillaume Carteaux^1,2^, MD, PhD, Armand Mekontso Dessap^1,2^ ,MD, PhD

**Correspondence and requests** should be addressed to Dr Keyvan Razazi, Service de Réanimation Médicale, CHU Henri Mondor, 51, Av de Lattre de Tassigny, 94000 Créteil Cedex, France. E-mail: [keyvan.razazi@aphp.fr](mailto:keyvan.razazi@aphp.fr) . Tel: 33.1.49.81.23.91; Fax: 33.1.49.81.49.43, ORCID number 0000-0001-7979-2650

**Definitions**

Failure of the SBT was defined as the development within one hour of any of the following: respiratory rate more than 35 breaths/min with increased accessory muscle activity, SpO_2_ (arterial oxygen saturation as determined by pulse oximetry) below 90% (with FiO_2_ ≥0.5 or an oxygen flow ≥10 L/min), heart rate >140 beats/min, systolic blood pressure <90 mm Hg or >180 mm Hg, major dyspnea or agitation, depressed mental status.

**Data collection**

For each patient, we collected demographics, comorbidities, Mac Cabe classification [class 0 denotes no underlying disease or non-fatal disease; class 1 denotes an ultimately fatal (1 to 5 years) underlying disease; class 2 denotes a rapidly (<1 year) fatal underlying disease], severity score at admission (Simplified Acute Physiologic Score II), reason for intubation, duration of mechanical ventilation, organ support, ICU length of stay, and outcome. ARDS was defined according to the Berlin definition [1]. The diagnosis of ventilator associated pneumonia was based on the following usual criteria: systemic signs of infection, new or worsening infiltrates on the chest roentgenogram, purulent tracheal secretions, and bacteriologic evidence of pulmonary parenchymal infection from distal airway sampling, preferably using a protected telescoping catheter or bronchoscopy and quantitative cultures (greater than or equal to 10^3^ and 10^4^ colony forming units/mL for protected telescoping catheter and bronchoalveolar lavage, respectively) [2].

**Lung ultrasonography and echocardiography**

Maximal end-expiratory interpleural distance, sonographic patterns of effusion (homogeneously anechoic, complex nonseptated*,* complex septated, or homogeneously echogenic) [3], and of lung parenchyma (condensation or atelectasis) [4] were assessed on each side with the patient in the semirecumbent position. A transthoracic echocardiography was also performed to assess left ventricle ejection fraction (computed from the bi-plane Simpson method [5] when image quality was good, or visually estimated when poor image quality did not allow sufficient identification of the endocardium) [6].

**References**

1. ARDS Definition Task Force, Ranieri VM, Rubenfeld GD, Thompson BT, Ferguson ND, Caldwell E, et al. Acute respiratory distress syndrome: the Berlin Definition. JAMA. 2012;307:2526–33.

2. Chastre J, Fagon J-Y. Ventilator-associated pneumonia. Am J Respir Crit Care Med. 2002;165:867–903.

3. Yang PC, Luh KT, Chang DB, Wu HD, Yu CJ, Kuo SH. Value of sonography in determining the nature of pleural effusion: analysis of 320 cases. AJR Am J Roentgenol. 1992;159:29–33.

4. Volpicelli G, Elbarbary M, Blaivas M, Lichtenstein DA, Mathis G, Kirkpatrick AW, et al. International evidence-based recommendations for point-of-care lung ultrasound. Intensive Care Med. 2012;38:577–91.

5. Lang RM, Bierig M, Devereux RB, Flachskampf FA, Foster E, Pellikka PA, et al. Recommendations for chamber quantification: a report from the American Society of Echocardiography’s Guidelines and Standards Committee and the Chamber Quantification Writing Group, developed in conjunction with the European Association of Echocardiography, a branch of the European Society of Cardiology. J Am Soc Echocardiogr Off Publ Am Soc Echocardiogr. 2005;18:1440–63.

6. Gudmundsson P, Rydberg E, Winter R, Willenheimer R. Visually estimated left ventricular ejection fraction by echocardiography is closely correlated with formal quantitative methods. Int J Cardiol. 2005;101:209–12.

**Table e1.** **Sonographic patterns of the pleural space and of lung parenchyma in 80 patients with moderate-to-large pleural effusion (one patient had missing data)**

| **Pleural effusion** |  |
| --- | --- |
| Homogeneously anechoic | 74 (93%) |
| Complex nonseptated | 4 (5%) |
| Complex septated | 2 (3%) |
| Homogeneously echogenic | 0 |
| **Lung parenchyma** |  |
| No condensation or atelectasis | 12 (15%) |
| Condensation | 48 (60%) |
| Atelectasis | 20 (25%) |

**Table e2. Fluid balance and change in interpleural distance according to fluid management during the 24 and 48 hours following failure of spontaneous breathing trial in 41 mechanically ventilated patients.**

|  | **Depletive fluid management during the 24 h following SBT failure** | | ***P***  ***value*** |
| --- | --- | --- | --- |
|  | **No (N=14)** | **Yes (n=27)** |  |
| Fluid balance at 24 hours after SBT (mL) | 858 [205 to 1806] | -484 [-1210 to 330] | 0.001 |
| Change in maximal interpleural distance at 24 hours after SBT (mm) | 0 [-2 to 0] | -0.5 [-10 to 0] | 0.12 |
|  | **Depletive fluid management during the 48 h following SBT failure** | | ***P***  ***value*** |
|  | **No (N=5)** | **Yes (n=24)** |  |
| Fluid balance at 48 hours after SBT (mL) | 1110 [-545 to 3357] | -160 [-2926 to 1025] | 0.09 |
| Change in maximal interpleural distance at 48 hours after SBT (mm) | 0 [0 to 0] | 0 [-10 to 0] | 0.19 |

Abbreviations: SBT= spontaneous breathing trial. Depletive fluid management denotes use of diuretic therapy or ultrafiltration. Change in maximal interpleural distance was computed by substracting the distance recorded at 24 or 48 hours to the distance recorded at spontaneous breathing trial.

**Table e3. Association of pleural effusion with weaning failure in sensitivity analyses**

| **Sensitivity analysis** | Number  of patients | Odd ratios [95% confidence interval] | P value |
| --- | --- | --- | --- |
| Adjustment on SAPS II | 246 | 3.2 (1.8-5.7) | p<0.001 |
| Selection of centers using the T-piece trial | 61 | 5.1 (1.5-17.6) | P=0.009 |
| Selection of centers using a low-level pressure support | 185 | 2.8 (1.4-5.3) | P=0.002 |
| Pleural effusions deemed drainable (as defined by a maximal interpleural distance ≥15 mm with the effusion visible over three intercostal spaces) | 246 | 2.5 (1.4-4.5) | P=0.002 |
| Pleural effusions considered large (as defined by a maximal interpleural distance ≥25 mm) | 246 | 3.8 (1.9-7.3) | P<0.001 |

**Table e4. Characteristics and outcome of 249 mechanically ventilated patients with or without moderate-to-large pleural effusion at first spontaneous breathing trial.**

| **Variables** | **Moderate-to-large pleural effusion** | |  |
| --- | --- | --- | --- |
|  | **Absent**  **(n=168)** | **Present**  **(n=81)** | ***P value*** |
| Male gender | 98 (58%) | 52 (64%) | 0.38 |
| Age (years) | 61 [50-72] | 69 [60-80] | <0.001 |
| Body mass index (kg/m^2^) | 26 [22-30] | 26 [22-31] | 0.53 |
| SAPS II score at ICU admission | 49 [37-62] | 52 [41-67] | 0.07 |
| **Comorbidities** |  |  |  |
| Mc Cabe class |  |  | <0.001 |
| 0 | 107 (64%) | 33 (41%) |  |
| 1 | 50 (30%) | 28 (35%) |  |
| 2 | 11 (7%) | 20 (25%) |  |
| COPD | 30 (18%) | 16 (20%) | 0.72 |
| Neurological disease | 22 (13%) | 6 (7%) | 0.18 |
| Cardiac disease | 93 (55%) | 65 (80%) | <0.001 |
| Systolic dysfunction | 25 (15%) | 20 (25%) | 0.06 |
| Diastolic dysfunction | 15 (9%) | 19 (24%) | 0.002 |
| Arrhythmia | 26 (16%) | 13 (16%) | 0.91 |
| Other | 79 (47%) | 55 (68%) | 0.002 |
| Cirrhosis | 12 (7%) | 9 (11%) | 0.29 |
| Chronic renal failure | 22 (13%) | 16 (20%) | 0.17 |
| Cancer or haematological malignancy | 16 (10%) | 22 (27%) | <0.001 |
| **Main reason for intubation** |  |  |  |
| Coma | 54 (32%) | 9 (11%) | <0.001 |
| Acute respiratory failure | 51 (30%) | 37 (46%) | 0.02 |
| Septic shock | 22 (13%) | 12 (15%) | 0.71 |
| Others | 41 (24%) | 23 (28%) | 0.5 |
| **From ICU admission to first SBT** |  |  |  |
| ARDS | 32 (19%) | 23 (28%) | 0.096 |
| Neuromuscular blockade | 34 (20%) | 21 (26%) | 0.31 |
| Septic shock | 62 (37%) | 40 (49%) | 0.06 |
| Steroids use | 51 (30%) | 26 (32%) | 0.78 |
| VAP | 20 (12%) | 13 (16%) | 0.37 |
| Supra-ventricular arrhythmias | 30 (18%) | 27 (33%) | 0.006 |
| Duration of MV before the first SBT | 4 [2-7] | 4 [3-9] | 0.09 |
| Dialysis | 15 (9%) | 16 (20%) | 0.015 |
| Fluid balance from ICU admission to first SBT (L) | 3 [0.9-7] | 5 [0.5-11] | 0.17 |
| **Biological and ultrasound data at first SBT** |  |  |  |
| PaO_2_/FiO_2_ ratio (mmHg) | 297 [230-376] | 270 [222-340] | 0.16 |
| Serum creatinine (µmol/L) | 74 [55-119] | 90 [60-164] | 0.07 |
| Serum protide (mg/L) | 59 [54-66] | 59 [51-63] | 0.19 |
| Bilateral pleural effusion | 16 (10%) | 60 (74%) | <0.001 |
| Maximal interpleural distance (mm) | 0 [0-5] | 27 [20-41] | <0.001 |
| Condensation or atelectasis of lung adjacent to the pleural effusion at ultrasound | - | 68 (84%) |  |
| Left ventricle ejection fraction at cardiac ultrasound (%) | 60 [50-60] | 50 [39-60] | <0.001 |
| **Outcome** |  |  |  |
| Pleural effusion drainage during weaning | 0 | 4 (5%) | 0.005 |
| Prophylactic NIV post extubation | 62 (38%) | 33 (43%) | 0.39 |
| Failure of the first SBT | 19 (11%) | 27 (33%) | <0.001 |
| Extubation failure | 24 (15%) | 15 (20%) | 0.31 |
| Weaning failure* | 36 (22%) | 37 (47%) | <0.001 |
| Weaning group** |  |  | 0.03 |
| Short weaning | 118 (70%) | 43 (53%) |  |
| Difficult weaning | 38 (20%) | 26 (32%) |  |
| Prolonged weaning | 16 (10%) | 12 (15%) |  |
| Tracheotomy | 4 (2%) | 2 (3%) | 0.97 |
| VFD from first SBT to day-28 (days) | 23 [16-26] | 21 [5-24] | 0.01 |
| Death in ICU | 14 (8%) | 13 (16%) | 0.07 |
| Death at day-28 | 14 (8%) | 14 (17%) | 0.04 |

* according to the international conference consensus (three patients could not be classified); ** according to the WIND study classification; abbreviations: SAPS II= simplified acute physiology score; COPD= chronic obstructive pulmonary disease; ARDS= acute respiratory distress syndrome; VAP= ventilator associated pneumonia; SBT= spontaneous breathing trial; NIV= non-invasive ventilation; ICU= intensive care unit; VFD= ventilator free days. Values are indicating number (%) or median [1^st^ - 3^rd^ quartile].

**Table e5. Variables associated with weaning failure in 246 mechanically ventilated patients** (**three patients could not be classified** **according to the international conference consensus definition).**

| **Variables** | **Weaning**  **success**  **(n=173)** | **Weaning**  **failure**  **(n=73)** | ***P value*** |
| --- | --- | --- | --- |
| Male gender | 98 (57%) | 49 (67%) | 0.13 |
| Age (years) | 61 [52-73] | 69 [60-79] | 0.006 |
| Body mass index (kg/m^2^) | 26 [22-29] | 27 [22-32] | 0.07 |
| SAPS II at ICU admission | 49 [38-62] | 49 [39-65] | 0.73 |
| **Comorbidities** |  |  |  |
| Mc Cabe class |  |  | 0.81 |
| 0  1  2 | 99 (57%)  51 (30%)  23 (13%) | 41 (56%)  24 (33%)  8 (11%) |  |
| COPD | 23 (13%) | 23 (32%) | 0.001 |
| Neurological disease | 21 (12%) | 7 (10%) | 0.59 |
| Cardiac disease | 101 (58%) | 55 (75%) | 0.01 |
| Systolic dysfunction | 26 (15%) | 19 (26%) | 0.04 |
| Diastolic dysfunction | 20 (12%) | 14 (19%) | 0.10 |
| Arrhythmia | 24 (14%) | 15 (21%) | 0.17 |
| Other | 87 (50%) | 45 (62%) | 0.11 |
| Cirrhosis | 15 (9%) | 5 (7%) | 0.56 |
| Chronic renal failure | 27 (16%) | 10 (14%) | 0.66 |
| Cancer or hematological malignancy | 22 (13%) | 15 (21%) | 0.14 |
| **Main reason for intubation** |  |  |  |
| Coma | 54 (31%) | 9 (12%) | 0.002 |
| Acute respiratory failure | 48 (27%) | 39 (53%) | <0.001 |
| Septic shock | 24 (14%) | 8 (11%) | 0.54 |
| Others | 47 (27%) | 17 (23%) | 0.53 |
| **From ICU admission to first SBT** |  |  |  |
| ARDS | 28 (16%) | 27 (37%) | <0.001 |
| Neuromuscular blockade | 26 (15%) | 28 (38%) | <0.001 |
| Septic shock | 61 (35%) | 39 (53%) | 0.01 |
| VAP | 17 (10%) | 16 (22%) | 0.01 |
| Supra-ventricular arrhythmias | 32 (19%) | 22 (30%) | 0.04 |
| Duration of MV before first SBT | 3 [2-6] | 6 [3-12] | <0.001 |
| Dialysis | 22 (13%) | 8 (11%) | 0.70 |
| Fluid balance between ICU admission and first SBT (L) | 2.8 [0.9-6.4] | 5.7 [0.7-11.4] | 0.01 |
| **Biological and ultrasound data at first SBT** |  |  |  |
| PaO_2_/FiO_2_ ratio (mmHg) | 307 [242-385] | 247 [200-299] | <0.001 |
| Creatininemia (µmol/L) | 76 [58-125] | 82 [56-132] | 0.94 |
| Serum Protide (mg/L) | 59 [53-64] | 59 [54-65] | 0.32 |
| Moderate to large pleural effusion | 42 (24%) | 37 (51%) | <0.001 |
| Drainable pleural effusion | 36 (21%) | 29 (40%) | 0.002 |
| Large pleural effusion | 21 (12%) | 25 (34%) | <0.001 |
| Left ventricle ejection fraction at cardiac ultrasound (%, n=205) | 60 [50-60] | 55 [40-60] | 0.06 |
| **Outcome** |  |  |  |
| Pleural effusion drainage during weaning | 0 | 4 (6%) | 0.01 |
| Prophylactic NIV post extubation | 65 (38%) | 30 (44%) | 0.35 |
| Tracheotomy | 1 (1%) | 5 (7%) | 0.01 |
| VFD from first SBT to day-28 (days) | 23 [20-26] | 11 [0-21] | <0.001 |
| Death in ICU | 5 (3%) | 19 (26%) | <0.001 |
| Death at day-28 | 8 (5%) | 17 (23%) | <0.001 |

Abbreviations: SAPS II= simplified acute physiology score; COPD= chronic obstructive pulmonary disease; ARDS= acute respiratory distress syndrome; VAP= ventilator associated pneumonia; SBT= spontaneous breathing trial; NIV= non-invasive ventilation; ICU= intensive care unit; VFD= ventilator free days.

Values are indicating number (%), or median [1^st^ - 3^rd^ quartile].

MacCabe class 0 = no underlying disease or non-fatal disease; class 1 = ultimately fatal (1 to 5 years) underlying disease; class 2 = rapidly (<1 year) fatal underlying disease.
