# Supplementary figures and images for: Pleural effusion during weaning from mechanical ventilation: a prospective observational multicenter study
Source: Ann Intensive Care. 2018 Nov 1;8:103. doi: 10.1186/s13613-018-0446-y (PMC6211142; doi:10.1186/s13613-018-0446-y)

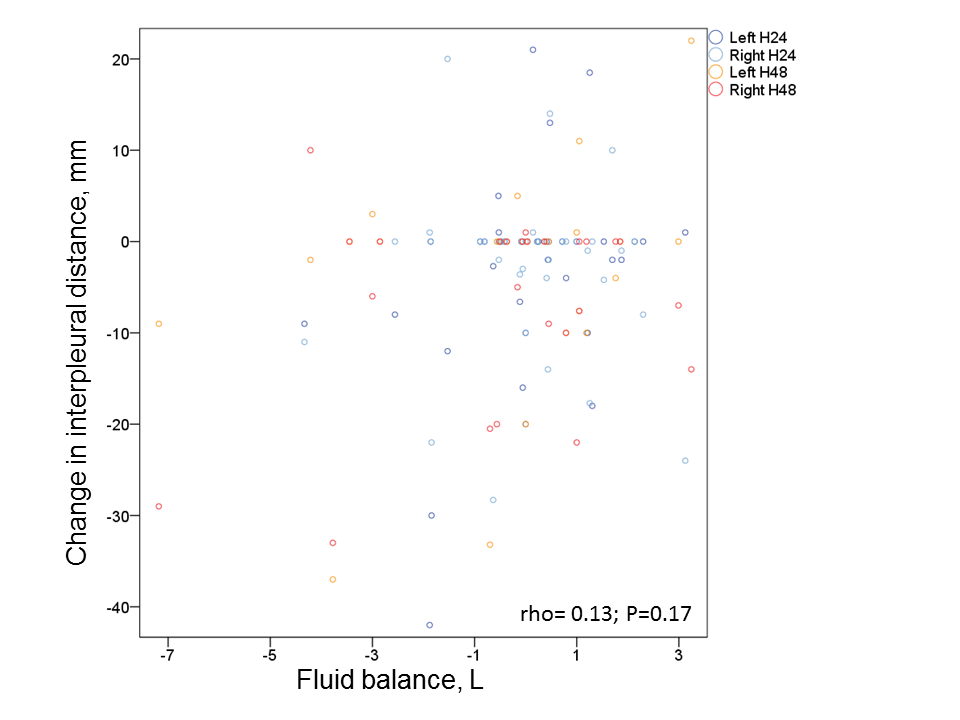

Supplement: Supplementary file 2 — Additional file 2. Change in interpleural distance during the 24 and 48 h following failure of spontaneous breathing trial according to fluid balance. [file 13613_2018_446_MOESM2_ESM.tif]
